# Supplementary material for: Dose-Dependent Outcome of EBV Infection of Humanized Mice Based on Green Raji Unit (GRU) Doses
Source: Viruses. 2021 Oct 29;13(11):2184. doi: 10.3390/v13112184 (PMC8624110; doi:10.3390/v13112184)
Supplement: Supplementary file 1 [file viruses-13-02184-s001.zip › viruses-1410138-supplementary.pdf]

Dose-dependent outcome of EBV infection of humanized mice based on Green Raji Unit (GRU) doses

Haiwen Chen, Ling Zhong, Wanlin Zhang, Shanshan Zhang, Junping Hong, Xiang Zhou, Xinyu Zhang, Qisheng Feng, Yixin Chen, Yi-Xin Zeng, Miao Xu, Claude Krummenacher, Xiao Zhang

Supplementary figures

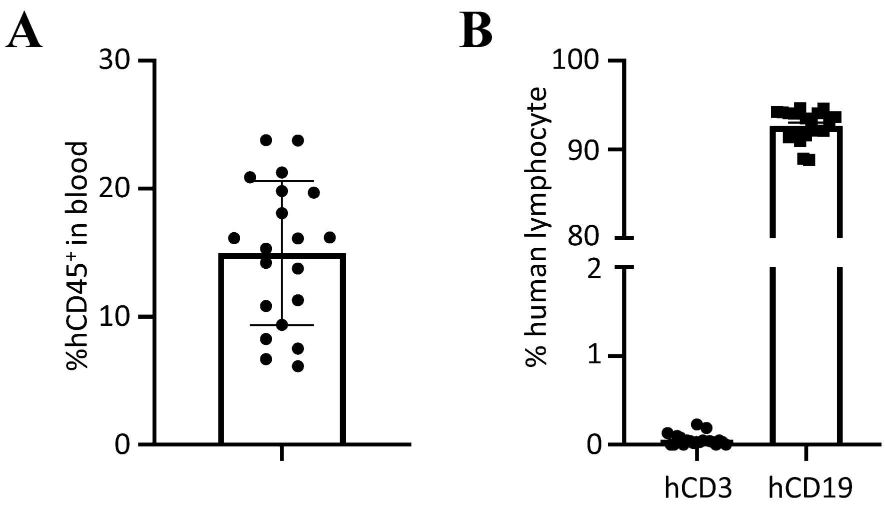

Figure S1. Frequency of human (h) hCD45⁺ (left) and, hCD19⁺ and hCD3⁺ cells (right) were measured 8 weeks following CD34⁺ stem cells engraftment but prior to viral challenge. Each dot represents a different mouse, n = 20.

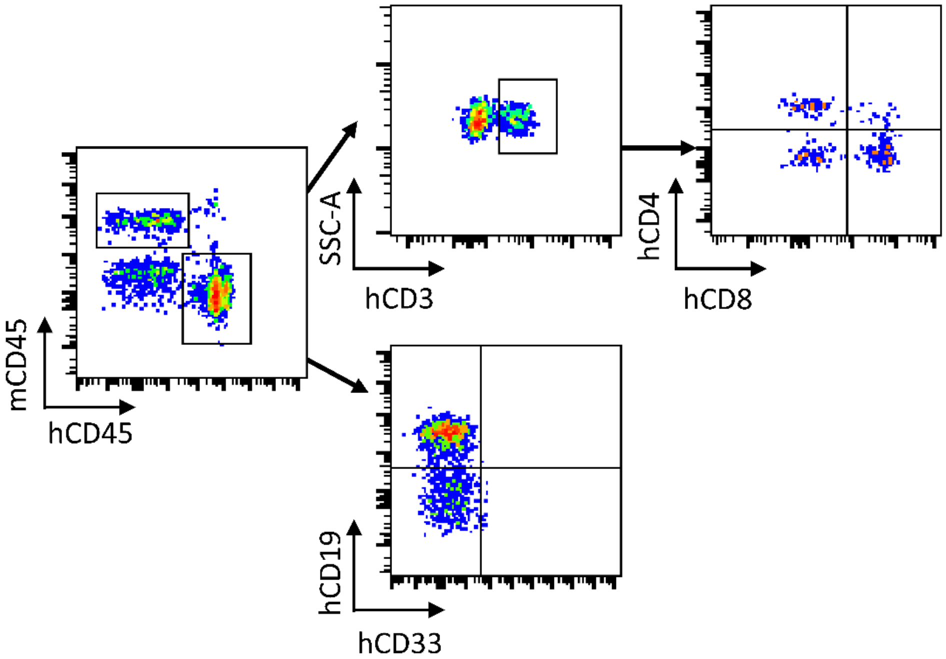

Figure S2. Representative flow plot showing the gating strategy used to analyze peripheral blood.

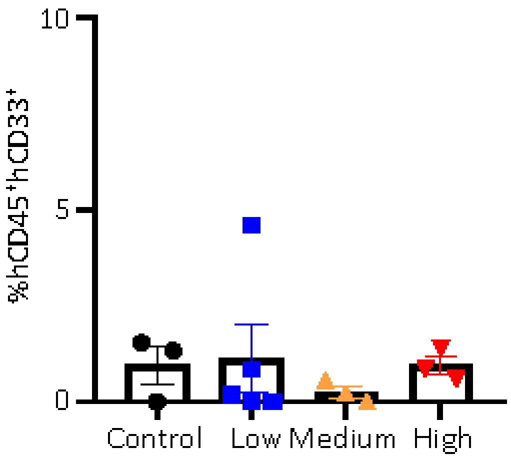

Figure S3. Splenic lymphocytes were analyzed in EBV-infected humanized mice. The frequency of hCD33^{+} myeloid cells in spleens at the study endpoint. Data points represent Mean ± SEM of uninfected control mice (n = 3), low (n = 5), medium (n = 3), high (n = 3) doses GRUs of Akata-EBV-GFP infected mice.

Supplementary table

Table S1. Primers for RT-PCR to detect EBV transcripts.

Primer	Sequence (5′→3′)
EBNA1	
Forward	aaggccatttttccaccctgtaggggatgc
Reverse	tcactcctgcccttcctcaccctcatctcc
EBNA2	
Forward	ggacacaagagccatcacctcttgataggg
Reverse	aatggcataggtggaatgttataaaataca
LMP2A	
Forward	atgaatccagtatgcctgcctgtaattgtt
Reverse	ccagaaggatcttggtcagtggacatgaag
LMP1	
Forward	tcatggaccacgacacactgatgaacacca
Reverse	caccggaaccagaagtacccaaaagcagcg
EBER1	
Forward	aggacctacgctgccctagaggttttgcta
Reverse	aaaacatgcggaccaccagctggtacttga
BZLF1	
Forward	atgatggacccaaactcgacttctgaagat
Reverse	ttagaaatttaagagatcctcgtgtaaaac
BMRF1	
Forward	ctagccgtcctgtccaagtgctatgaccat
Reverse	cgtggtgacgtagagatccggattgagtgt
BLLF1	
Forward	agccttgcttgtgtgtcagtacaccatcca
Reverse	ggcaccacatggttccaaacacatcttgaa
GAPDH	
Forward	gcctcctgcaccaccaactg
Reverse	cgacgcctgcttcaccaccctct
